# Application of a posterior tibial artery perforator-based fasciocutaneous flap with a broad fascial pedicle in reconstruction of complex lower-leg wounds: a retrospective clinical study

**DOI:** 10.3389/fsurg.2026.1849803

**Published:** 2026-06-24

**Authors:** Mingju Gao, Zeming Lang, Haibo Li, Xinhui Du

**Affiliations:** 1 The First Affiliated Hospital of Shihezi University, Shihezi, Xinjiang, China; 2 Department of Trauma Orthopedics, The First Affiliated Hospital of Shihezi University, Shihezi, Xinjiang, China

**Keywords:** broad fascial pedicle, complex lower-leg wound, fasciocutaneous flap, lower extremity reconstruction, pedicled perforator flap, posterior tibial artery perforator flap

## Abstract

**Background:**

Complex soft-tissue defects of the lower leg with exposed bone, tendon, or internal fixation material remain difficult to reconstruct, particularly when the surrounding wound bed has been affected by infection, scar formation, or previous surgery. Free flaps are reliable for extensive defects but require microsurgical expertise and suitable recipient vessels. This study evaluated the clinical outcomes of a posterior tibial artery perforator-based fasciocutaneous flap with a broad fascial pedicle for selected complex lower-leg wounds, while preserving the main posterior tibial artery.

**Methods:**

We retrospectively reviewed 15 patients (11 males and 4 females; mean age, 43.5 years; range, 22–65 years) who underwent reconstruction of complex medial or distal lower-leg wounds between January 2019 and January 2024. Etiologies included post-traumatic tissue loss (*n* = 8) and chronic post-surgical wounds after osteomyelitis debridement (*n* = 7). Preoperative handheld Doppler mapping was used to localize posterior tibial artery perforators and guide flap design. Surgical outcomes, flap viability, donor-site morbidity, sensory recovery using the Medical Research Council scale, and ankle motion were assessed during 6–24 months of follow-up.

**Results:**

All 15 flaps survived completely, with no partial or total necrosis. No hematoma, infection beneath the flap, donor-site functional deficit, or revision surgery occurred. At final follow-up, 8 flaps recovered to S3, 5 to S2, and 2 to S1. Active ankle dorsiflexion and plantarflexion were preserved in all patients, and all patients regained independent ambulation. Donor-site morbidity was minimal, with acceptable linear scarring or healed split-thickness skin grafts.

**Conclusion:**

In this single-center retrospective clinical study, a posterior tibial artery perforator-based fasciocutaneous flap with a broad fascial pedicle provided stable coverage for selected complex medial and distal lower-leg wounds. This technique should be interpreted as one application within the established spectrum of posterior tibial artery perforator-based flaps rather than as a distinct new flap type. Because this study was retrospective, uncontrolled, and limited by a small sample size, prospective comparative studies are required to define its relative indications and outcomes compared with other reconstructive options.

## Introduction

Lower-leg soft-tissue defects, particularly those located on the medial aspect or around the ankle, represent an important reconstructive challenge. The medial lower leg has a thin soft-tissue envelope overlying the tibia and tendons, leaving bone, tendon, or internal fixation material vulnerable to exposure after trauma, infection, repeated debridement, or orthopedic surgery. Such wounds often fail to heal with conventional dressings or skin grafting alone because the wound bed may have poor vascularity, persistent dead space, bacterial contamination, or inadequate soft-tissue coverage. Durable flap coverage is therefore frequently required to introduce well-vascularized tissue, protect deep structures, and enable limb salvage (1, 2).

Two broad reconstructive strategies are commonly considered for these wounds: free tissue transfer and pedicled local or regional flaps. Free flaps provide a large volume of vascularized tissue and remain essential for extensive composite defects, particularly when local tissues are unavailable. However, free flap reconstruction requires microsurgical anastomosis, an experienced team, and suitable recipient vessels. In selected moderate-sized defects, pedicled perforator flaps may avoid microvascular anastomosis while providing local tissue replacement (3, 4).

Pedicled flaps avoid distant vascular anastomosis and can be attractive when the defect is moderate in size and when reliable local blood supply remains available. Nevertheless, perforator-based propeller and regional pedicled flaps can be associated with venous congestion, marginal necrosis, or delayed healing, particularly when the flap is long, the arc of rotation is large, or the surrounding tissue has been affected by scar or previous infection (5, 6).

Posterior tibial artery perforator-based flaps offer several anatomical and practical advantages for medial lower-leg and ankle defects. Anatomical studies have shown that the posterior tibial artery gives off repeated perforators along the medial leg (7, 8). These perforators are related to suprafascial vascular pathways, venous channels, and superficial sensory nerves that are relevant to flap planning (9, 10). More recent anatomical work has further supported the importance of understanding posterior tibial artery perforator distribution when designing propeller or perforator-based flaps (11). In the present series, the flap was designed as a posterior tibial artery perforator-based fasciocutaneous flap with a broad fascial pedicle. The technical feature emphasized was preservation of a broad fascial sleeve containing serial perforators and fascial vascular interconnections, rather than skeletonization of a single perforator or introduction of a distinct new flap nomenclature.

Posterior tibial artery perforator-based adipofascial, fasciocutaneous, propeller, and distally based flap variants have been previously described for reconstruction of distal leg, ankle, and foot defects (12–14). Distally based posterior tibial artery flaps and propeller-type posterior tibial perforator flaps have also been reported for selected ankle, Achilles-region, and lower-leg defects (15–17). Clinical series have reported posterior tibial perforator-based flaps as an option for selected leg, ankle, and foot defects, while emphasizing indications and technical limitations (18, 19). Therefore, the present study does not aim to introduce a new flap type. Rather, it reports our clinical experience using a posterior tibial artery perforator-based fasciocutaneous flap with a broad fascial pedicle in 15 selected patients with complex medial or distal lower-leg wounds. The objective of this Original Research article was to evaluate flap survival, donor-site morbidity, sensory recovery, ankle function, and clinically observed wound healing, and to discuss the anatomical rationale, technical considerations, and limitations of this vessel-preserving approach within the established family of posterior tibial artery perforator-based flaps.

## Materials and methods

### Study design and ethical approval

This was a retrospective, single-center observational clinical study. Medical records and follow-up data were reviewed for patients who underwent posterior tibial artery perforator-based fasciocutaneous flap reconstruction with a broad fascial pedicle for complex lower-leg wounds at our institution from January 2019 to January 2024. The study was approved by the Science and Technology Ethical Committee of the First Affiliated Hospital of Shihezi University (approval No. KJ2026-061-02). The requirement for informed consent for retrospective data analysis was waived by the ethics committee. Written informed consent was obtained for publication of identifiable clinical images.

### Patients and wound characteristics

Fifteen patients met the study criteria. There were 11 males and 4 females, with ages ranging from 22 to 65 years and a mean age of 43.5 years. Defect etiologies included post-traumatic soft-tissue loss with exposed bone or tendon in 8 patients and chronic post-surgical wounds after osteomyelitis debridement in 7 patients. The wounds were located on the middle to lower medial leg or around the medial ankle. Defect sizes varied, with a maximum reported wound size of approximately 5 × 8 cm. All wounds had exposure of at least one deep structure, such as bone, tendon, or internal fixation material, and most patients had a history of infection or previous surgery. Baseline demographic and wound characteristics are summarized in Table 1. De-identified patient-level baseline and wound variables are provided in Supplementary Table S1.

**Table 1:** Baseline patient and wound characteristics of the study cohort (*n* = 15).

Characteristic	Value
Patients	15
Sex	11 male, 4 female
Age (years)	Mean 43.5 (range, 22–65)
Wound etiology	Post-traumatic defects with exposed bone/tendon (*n* = 8); chronic post-surgical wounds after osteomyelitis debridement (*n* = 7)
Wound location	Medial middle to distal lower leg and ankle
Maximum wound size	Up to 5 × 8 cm
Defect depth	All with exposed bone and/or tendon
Time from injury to reconstruction	More than 6 weeks in all cases
History of infection or prior surgery	Present in most patients
Preoperative wound management	Antibiotics with or without negative-pressure wound therapy when clinically indicated

**Figure 1:**
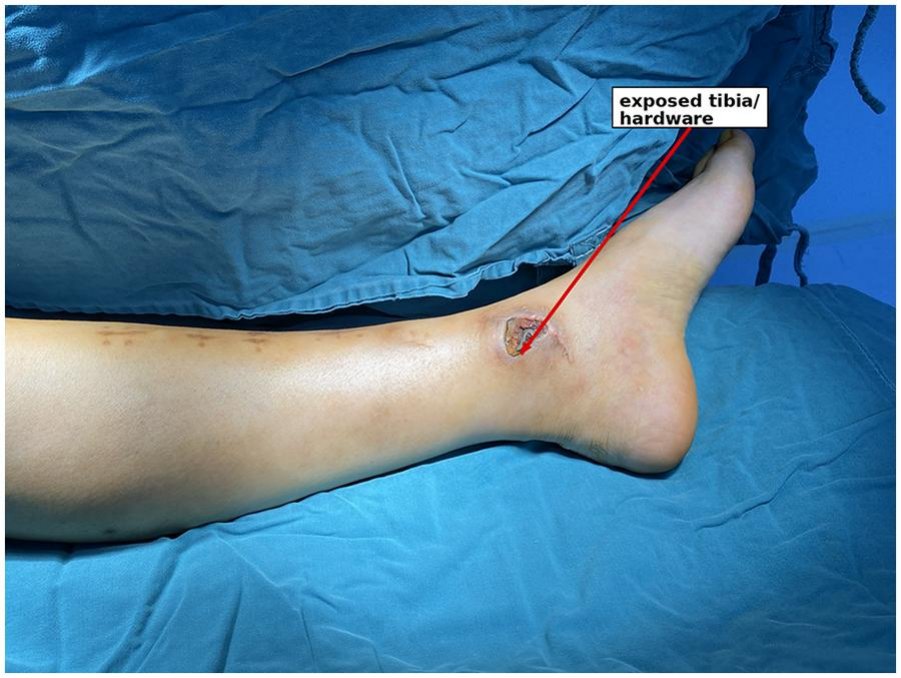
Preoperative appearance of the complex wound on the left medial malleolus with exposed tibial bone and internal fixation material. The red arrow indicates the exposed tibia/hardware within the defect. The wound measured 4.0 × 5.0 cm and required flap coverage.

**Figure 2:**
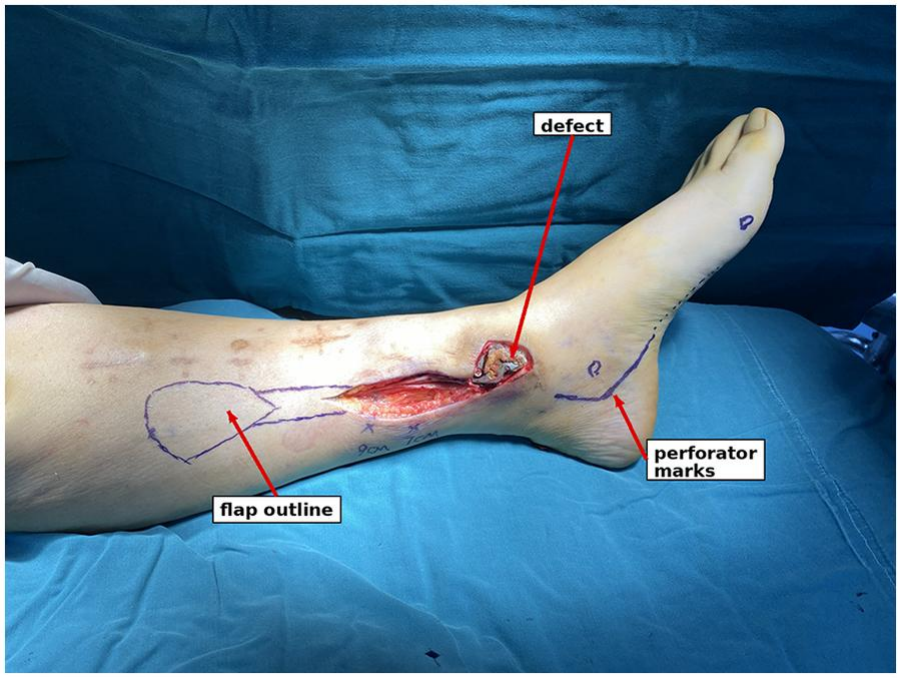
Preoperative design of the posterior tibial artery perforator-based fasciocutaneous flap with a broad fascial pedicle on the medial leg. The labels indicate the wound defect, flap outline, and Doppler-marked perforator locations along the medial-leg axis.

**Figure 3:**
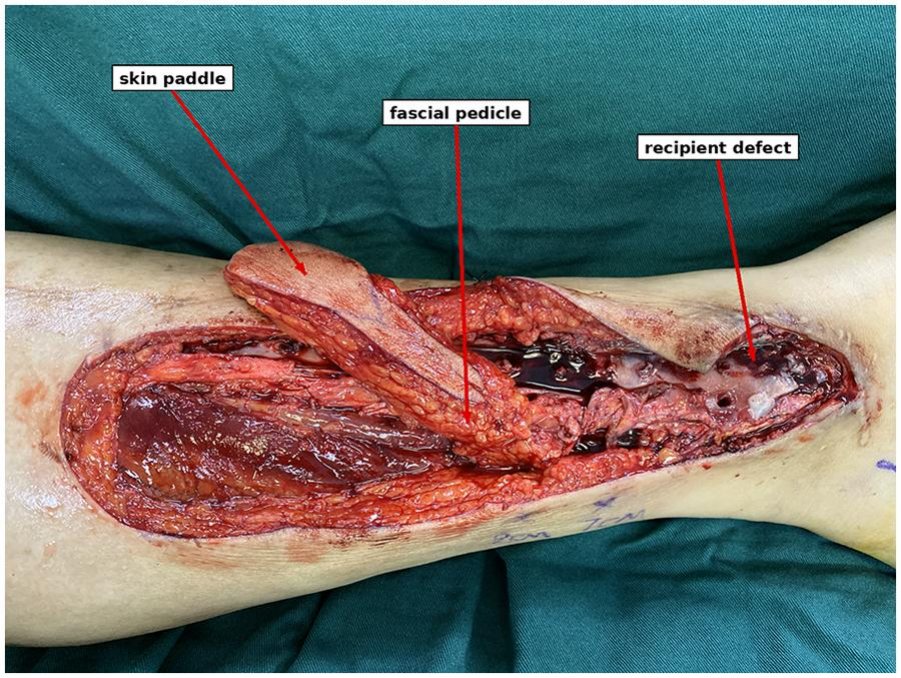
Intraoperative elevation of the posterior tibial artery perforator-based fasciocutaneous flap with a broad fascial pedicle. The labels indicate the skin paddle, broad fascial pedicle, and recipient defect. The flap was raised in the subfascial plane while preserving serial posterior tibial artery perforators and fascial vascular interconnections within the pedicle.

**Figure 4:**
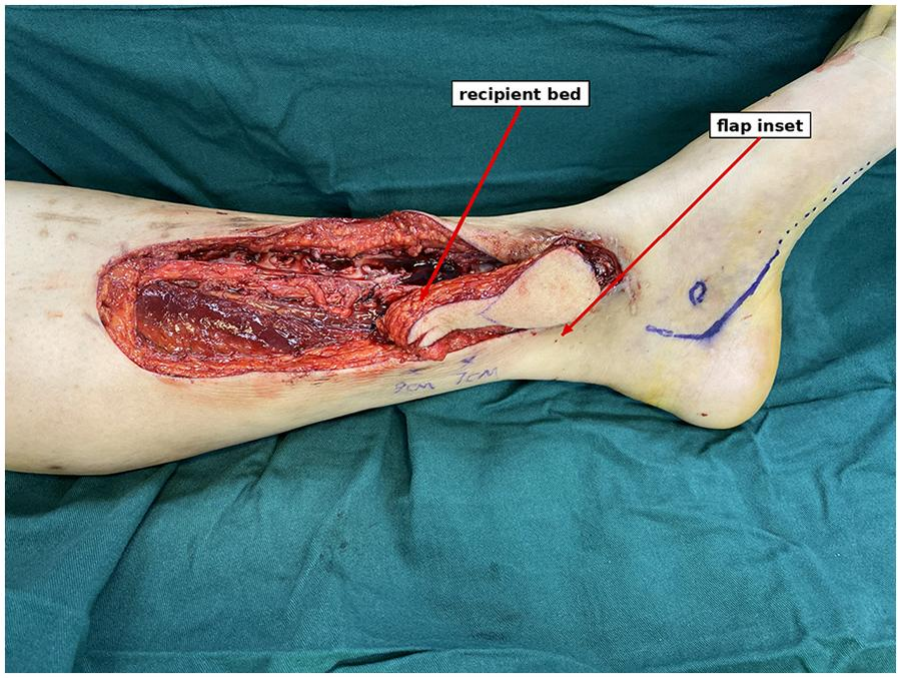
Transfer and inset of the broad-pedicle posterior tibial artery perforator-based flap to the medial ankle defect. The labels identify the recipient bed and flap inset. The flap was tunneled and inset without tension to cover the exposed tibia and internal fixation material.

**Figure 5:**
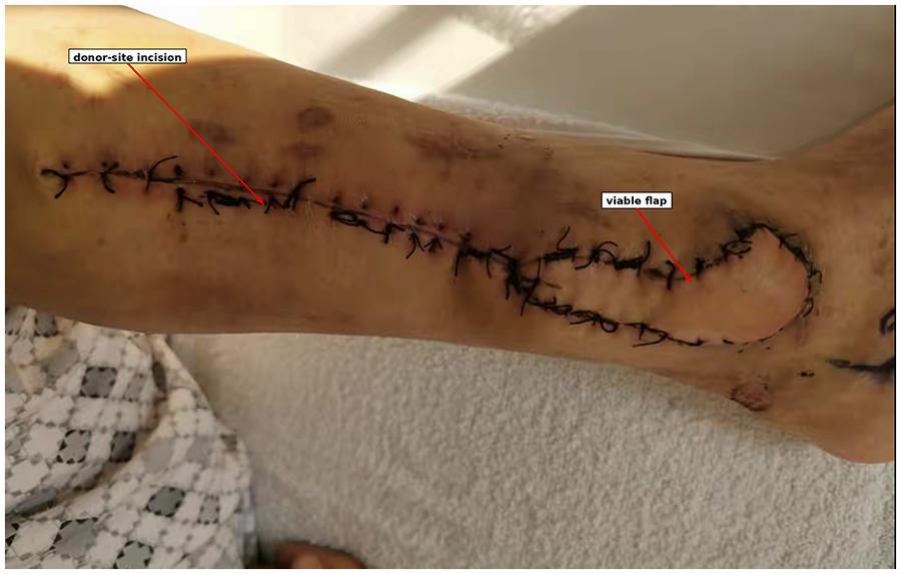
Postoperative appearance at 2 weeks. The labels indicate the viable flap and donor-site incision. The flap showed healthy color without necrosis, and the donor site showed satisfactory early healing.

**Figure 6:**
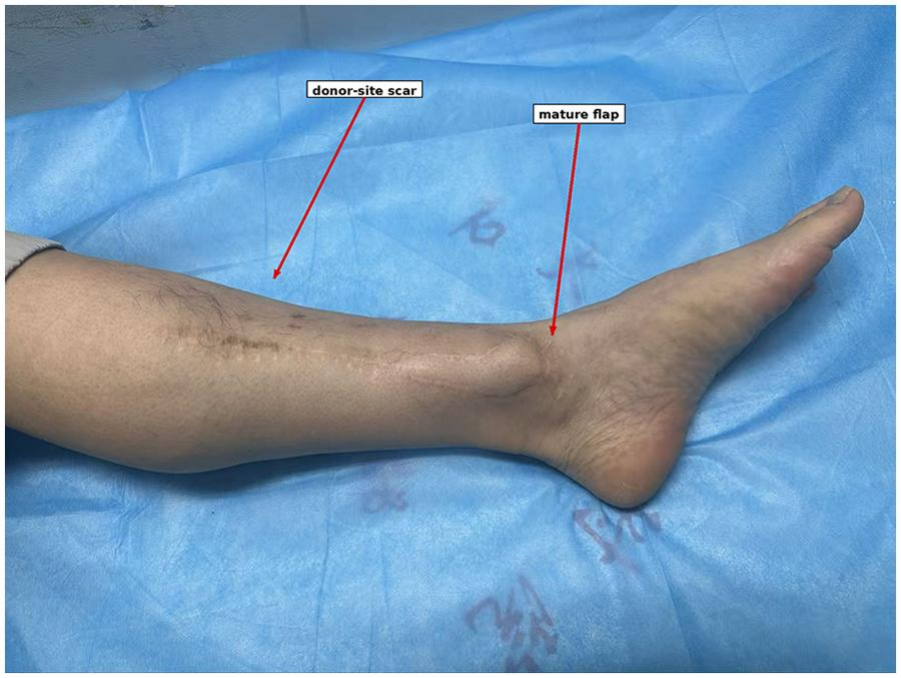
Long-term outcome at 2 years after surgery. The labels indicate the mature flap and donor-site scar. The flap remained durable and pliable with a contour similar to surrounding skin, and the medial-leg donor site showed a faint linear scar.

Because this was a retrospective study, the availability of serial clinical photographs varied across patients. Complete serial photographs suitable for publication were available for the representative case shown in Figures 1–6. To improve transparency beyond this representative case, de-identified patient-level baseline, operative, and outcome data for all 15 patients are provided in Supplementary Table S1.

Values are presented as number of patients unless otherwise indicated.

For the purpose of this study, a complex lower-leg wound was defined as a defect between the knee and ankle that required flap coverage because of exposed bone, tendon, hardware, or an inadequately vascularized wound bed, and that was not considered suitable for simple skin grafting alone. This operational definition was used to avoid ambiguity in terminology and to make the clinical indications clear. The term does not imply that all lower-leg wounds of this type should be treated with the same flap; rather, it defines the subgroup of patients treated in this series.

### Inclusion and exclusion criteria

The inclusion criteria were as follows: (1) a complex medial or distal lower-leg wound requiring flap coverage; (2) exposure of bone, tendon, or hardware, or deficient soft-tissue coverage after debridement; (3) reconstruction using a posterior tibial artery perforator-based fasciocutaneous flap with a broad fascial pedicle; and (4) available clinical and follow-up records. The exclusion criteria were as follows: (1) general condition unsuitable for surgery; (2) inability to tolerate anesthesia or postoperative limb positioning; (3) a wound requiring a different reconstructive strategy because of extent, location, or absence of usable local tissue; and (4) unavailable follow-up information. Chronic infection and medical comorbidities were not considered absolute exclusions if the wound could be optimized before flap transfer.

### Preoperative vascular and wound assessment

All patients underwent clinical assessment of the affected limb, including inspection of the wound, surrounding scar, skin quality, edema, and evidence of infection. The presence of exposed bone, tendon, or hardware was documented. Preoperative handheld Doppler mapping was performed along the medial leg to identify audible posterior tibial artery perforator signals and to assist in determining the axis, pivot point, and reach of the planned flap. Particular attention was paid to identifying a healthy region adjacent to the wound where the serial posterior tibial artery perforators and fascial vascular connections were likely to remain intact. Because the study was retrospective, additional vascular imaging was not uniformly available for all patients and therefore was not analyzed as a standardized outcome. The absence of standardized angiographic assessment is recognized as a limitation of this study.

Before definitive reconstruction, wounds with suspected or established infection were optimized by debridement, antibiotic treatment, and negative-pressure wound therapy when clinically indicated. The goal of preoperative wound management was to remove necrotic tissue, reduce bacterial burden, improve the wound bed, and create stable conditions for flap transfer. Although detailed microbiological data were not uniformly available for all patients in the original dataset, chronic osteomyelitis or infection history was clinically relevant in a substantial proportion of the cohort. This point was considered when interpreting the results and when formulating the limitations of the study.

### Flap design and surgical technique

All patients underwent a point-line-plane three-dimensional design protocol. The pivot point or flap pedicle was first identified in healthy tissue near the wound, often at or slightly above the distal perforator plexus located approximately 3–5 cm above the medial malleolus. A longitudinal axis line was then drawn along the medial border of the tibia, corresponding to the expected course of posterior tibial artery perforators. The skin paddle was outlined symmetrically around this line and was designed slightly larger than the defect, generally by approximately 10%–20%, to allow tension-free inset and account for soft-tissue contraction. When needed, the flap was extended proximally along the axis to recruit additional serial perforators. A safety margin beyond the most distal planned perforator was also preserved to reduce the risk of inadequate perfusion.

Under tourniquet control, the flap was elevated in a subfascial plane. A 3–4 cm wide strip of deep fascia was preserved with the flap to form a broad fascial pedicle. The flap was incised through the skin and subcutaneous tissue to the fascia, and blunt dissection was then used to elevate the flap while deliberately keeping the fascia attached to the skin paddle. This maneuver allows the fascial sleeve to carry serial posterior tibial artery perforators and associated small vascular branches. Individual perforators were not skeletonized, because the aim of this technique was to preserve the longitudinal fascial vascular network rather than isolate a single perforator. When a sensory nerve branch was encountered and could be preserved safely, it was included in the flap; nerve coaptation was performed in selected cases according to recipient-site conditions and surgeon judgment.

After elevation, the flap was transferred to the defect by rotation, advancement, or passage through a gentle subcutaneous tunnel. The pedicle was checked carefully to avoid kinking, compression, or excessive tension. If the tunnel was tight, the incision was extended to protect the pedicle. The flap was inset to cover exposed bone, tendon, or hardware with well-vascularized tissue. No microsurgical vascular anastomosis was required. The donor site was closed primarily when the flap width permitted tension-free approximation, particularly for flaps with a width of 7 cm or less. For larger donor defects, split-thickness skin grafting from the thigh was used, and negative-pressure therapy was applied to secure grafts when required.

### Postoperative care and follow-up

Postoperative care focused on maintaining flap perfusion, preventing venous congestion, controlling infection risk, and restoring limb function. The affected limb was elevated approximately 30 degrees to promote venous return and reduce edema. Local warming was used when appropriate to maintain a favorable temperature for microcirculation during the early postoperative period. Patients received postoperative medications for 7–10 days according to clinical need, including antibiotic prophylaxis or treatment, anticoagulants, and vasodilator or antispasmodic agents. Flap circulation was monitored closely during the first 48 h by observing skin color, capillary refill, temperature, swelling, and bleeding response. No vascular crisis requiring revision was recorded in this cohort.

Sutures were removed at approximately 14 days after surgery if healing was satisfactory. Gentle ankle range-of-motion exercises were encouraged after the early healing period to reduce stiffness, particularly in patients with wounds near the ankle. Weight-bearing was delayed until flap stability and wound healing were considered adequate, usually after at least 3–4 weeks. Patients were followed at scheduled intervals after surgery, including 1, 3, 6, 12, and 24 months when available. Follow-up duration ranged from 6 to 24 months, with a mean of approximately 14 months.

### Outcome assessment

The primary clinical outcome was flap survival, including complete survival and the presence or absence of partial or total flap necrosis. Secondary outcomes included wound healing, hematoma, infection beneath the flap, venous congestion, donor-site morbidity, need for secondary revision surgery, sensory recovery, ankle motion, and independent ambulation. Sensory recovery was assessed using the Medical Research Council sensory grading system, with S0 indicating no recovery, S1 deep pressure sensation, S2 some superficial pain and touch, S3 improved protective sensation, and S4 normal sensation. Active ankle dorsiflexion and plantarflexion angles were recorded in degrees. Because this was a retrospective study, standardized patient-reported outcome measures, validated functional scores, and serial imaging data were not available for all patients.

### Statistical analysis

Because of the small sample size and the descriptive nature of this retrospective clinical study, no comparative statistical testing was performed. Continuous variables are reported as means and ranges when available, and categorical variables are reported as numbers and percentages. The study was not designed or powered to compare this posterior tibial artery perforator-based fasciocutaneous flap with a broad fascial pedicle with free flaps, sural flaps, peroneal perforator flaps, propeller flaps, or other reconstructive options.

## Results

### Patient and wound profile

The study cohort included 15 patients with complex medial or distal lower-leg wounds. The demographic and wound characteristics are summarized in Table 1. Post-traumatic soft-tissue defects accounted for 8 cases, and chronic post-surgical wounds after osteomyelitis debridement accounted for 7 cases. Wounds were located from the middle medial lower leg to the medial ankle region. All cases required flap coverage because of exposed bone, tendon, or hardware, or because the wound bed was not suitable for simple skin grafting alone. Most patients had a history of infection, previous surgery, or local scar formation, which increased the difficulty of reconstruction.

### Flap survival and wound healing

All 15 posterior tibial artery perforator-based fasciocutaneous flaps with a broad fascial pedicle survived completely, yielding a flap survival rate of 100%. No patient developed total flap loss or marginal partial necrosis. The postoperative course was generally uneventful, with no hematoma or infection beneath the flap recorded in the original dataset. At final follow-up, all reconstructed wounds were healed. The flaps adapted well to the recipient sites, providing durable soft-tissue coverage over previously exposed structures. The skin paddle generally matched the surrounding skin in color and texture sufficiently for a functional lower-leg reconstruction, and the flap tissue remained pliable without excessive bulk.

Donor sites also healed satisfactorily. When primary closure was possible, the donor site resulted in a linear scar along the medial leg. When split-thickness skin grafting was used, the grafted donor area healed with acceptable contour and no functional deficit. No patient required revision surgery for either the recipient site or the donor site. Overall surgical outcomes and postoperative functional results are summarized in Table 2. De-identified patient-level operative variables and postoperative outcomes are provided in Supplementary Table S1.

**Table 2:** Surgical outcomes and postoperative functional results following posterior tibial artery perforator-based fasciocutaneous flap reconstruction with a broad fascial pedicle.

Outcome	Result
Flap survival	15/15 (100%)
Partial or total flap necrosis	None
Follow-up duration	6–24 months (mean approximately 14 months)
Sensory recovery (MRC scale)	S3: 8 patients; S2: 5 patients; S1: 2 patients
Ankle dorsiflexion	15 degrees–30 degrees
Ankle plantarflexion	30 degrees–45 degrees
Ambulation	Independent ambulation in all patients
Donor-site closure	Primary closure or split-thickness skin graft according to flap width and tension
Donor-site morbidity	Minimal; no functional deficits recorded
Postoperative complications	None recorded
Revision surgery	None
Microsurgical anastomosis	Not required

MRC = Medical Research Council sensory grading system.

### Sensory recovery and ankle function

Most patients regained some degree of protective sensation in the transferred flap. According to the Medical Research Council sensory grading system, 8 patients recovered to S3, 5 patients recovered to S2, and 2 patients recovered to S1 by the final follow-up. None reached S4 at the last follow-up, which may reflect the limited follow-up duration in some patients and the expected slow pace of sensory recovery in reconstructed lower-leg skin. These sensory findings suggest that the flap can provide useful protective sensation in many patients, although the retrospective design and lack of standardized quantitative sensory testing prevent stronger conclusions.

Ankle motion was preserved in all patients. At final assessment, active ankle dorsiflexion ranged from 15 degrees to 30 degrees, and plantarflexion ranged from 30 degrees to 45 degrees. All patients were able to ambulate independently. No obvious gait instability or donor-site functional deficit was documented. Because preoperative ankle motion and validated functional outcome scores were not uniformly recorded, these findings should be interpreted as descriptive clinical observations rather than as proof of superior functional recovery compared with other reconstructive methods.

### Representative case

A representative case illustrates the clinical application of the technique. A 45-year-old male patient had a chronic non-healing wound over the left medial ankle after open tibial fracture fixation. Six months after injury, he presented with a 4.0 cm × 5.0 cm defect with exposed tibial bone and internal fixation material (Figure 1). A 5.0 cm × 6.0 cm distally based posterior tibial artery perforator-based fasciocutaneous flap with a broad fascial pedicle was designed adjacent to the wound after handheld Doppler mapping of perforator locations (Figure 2). Similar use of posterior tibial artery perforator-based tissue for medial malleolar defects with exposed bone or hardware has been reported previously (20). The flap was elevated from the distal medial leg in a subfascial plane, preserving serial posterior tibial artery perforators and fascial vascular interconnections (Figure 3), and was tunneled medially to cover the defect. After inset, the flap fully covered the exposed bone and hardware without tension (Figure 4).

The postoperative course was uneventful. At 2 weeks, the flap was viable, no necrosis was observed, and the donor site was healing well (Figure 5). At 6 months, the flap had regained protective sensation, and ankle motion was clinically satisfactory. At 2 years, the reconstruction remained durable; the flap was pliable, the contour was close to the surrounding skin, and the donor-site scar was minimal (Figure 6). The patient returned to daily activities without recorded complications.

## Discussion

### Principal findings

This retrospective clinical study reviewed 15 patients who underwent posterior tibial artery perforator-based fasciocutaneous flap reconstruction with a broad fascial pedicle for complex medial or distal lower-leg wounds. All flaps survived completely, and no partial necrosis, total necrosis, hematoma, infection beneath the flap, donor-site functional deficit, or revision surgery was recorded. Most patients achieved at least partial sensory recovery, and active ankle dorsiflexion and plantarflexion were preserved within a functional range. These results suggest that this vessel-preserving posterior tibial artery perforator-based flap may be a feasible local reconstructive option in selected patients. However, the findings must be interpreted cautiously because the study was single-center, retrospective, uncontrolled, and limited to 15 cases.

### Clinical rationale for using a posterior tibial artery perforator-based fasciocutaneous flap with a broad fascial pedicle

Complex lower-leg wounds require reconstruction that can satisfy several conditions simultaneously. First, the transferred tissue must be sufficiently vascularized to cover exposed bone, tendon, or hardware and to improve the local wound environment. Second, the technique should minimize additional morbidity, particularly in patients who may already have compromised limb circulation or previous operations. Third, the reconstruction must tolerate motion and loading after healing, because the lower leg and ankle are functionally demanding regions. A posterior tibial artery perforator-based fasciocutaneous flap with a broad fascial pedicle is designed to address these requirements by using adjacent medial-leg tissue while preserving the main posterior tibial artery.

The anatomical logic of the broad-pedicle design differs from that of a highly skeletonized single-perforator propeller flap. In a single-perforator design, the entire flap depends heavily on one selected perforator and its capacity to perfuse the full skin paddle after rotation. This approach is precise and often effective, but it may be vulnerable when the chosen perforator is small, injured, scarred, or anatomically variable. In contrast, the present design preserves a broader fascial pedicle containing serial posterior tibial artery perforators and their fascial vascular connections. The goal is not to isolate one vessel but to maintain a local vascular network that can distribute inflow and outflow along the flap. This is particularly relevant in wounds with scarring or previous infection, where local perforator anatomy may be less predictable.

The posterior tibial artery is also anatomically favorable for medial lower-leg and ankle reconstruction because it runs in the same general region as many medial defects. The flap can therefore be designed adjacent to the wound, avoiding the need to transpose tissue across distant vascular territories. In addition, the donor-site scar is located on the medial leg and may be less conspicuous than scars created by some alternative donor sites. The technique does not require microsurgical anastomosis, which may shorten operative time and make reconstruction more accessible in centers where free flap transfer is not always available. These practical advantages help explain why a posterior tibial artery perforator-based flap may be useful for selected complex wounds.

### Evolution and anatomical basis of the technique

The concept of posterior tibial artery-based reconstruction has evolved over time. Earlier medial-leg fasciocutaneous flaps often depended on the main posterior tibial artery, and some designs required sacrificing or temporarily interrupting a major axial vessel. Although these approaches could provide coverage, they raised concern about distal limb perfusion and were not ideal for patients with compromised vascular reserve. Later neurocutaneous and adipofascial flap designs attempted to preserve the main artery by relying on the saphenous neurovascular network, venous plexuses, or small perforating vessels. These methods expanded the reconstructive options for lower-leg defects, but flap size, arc of rotation, and perfusion reliability remained important considerations.

The development of perforator flap surgery shifted attention toward preserving source vessels while using discrete perforators to supply local or regional tissue. Single posterior tibial artery perforator flaps, propeller flaps, adipofascial flaps, fasciocutaneous flaps, and distally based variants have been previously described for selected ankle, foot, and lower-leg defects. Their precision is attractive, because tissue harvest can be tailored around a mapped perforator. However, in complex wounds, the single selected perforator may be close to the zone of injury, surrounded by scar, or affected by infection. If that perforator is damaged or if the flap is designed beyond its reliable vascular territory, distal ischemia or venous congestion may occur. Techniques intended to improve venous reliability or preserve a broader neurovascular network have therefore been described in the posterior tibial artery perforator flap literature (21, 22). This concern provided part of the rationale for preserving a broader fascial pedicle in the present cohort.

The terminology in this revised manuscript was deliberately adjusted to avoid implying that the technique represents a completely new flap type. The present series should be interpreted as a clinical application within the established spectrum of posterior tibial artery perforator-based flaps. The technical feature emphasized in our cohort was preservation of a broad fascial pedicle containing serial perforators and fascial vascular interconnections, rather than complete skeletonization of a single perforator. Anatomical studies of the medial lower leg have described repeated perforators from the posterior tibial artery and communicating vascular branches within the deep fascia and subcutaneous tissue (6–8). These connections may support a longitudinal fasciocutaneous territory, allowing adjacent perforator zones to contribute to flap perfusion. The present clinical study was not an anatomical investigation and did not directly measure vascular flow; therefore, the anatomical explanation is offered as the rationale for flap design rather than as a conclusion proven by this dataset.

Venous drainage should also be considered. Large or long pedicled flaps may fail not only from inadequate arterial inflow but also from impaired venous outflow. Preserving a broad fascial pedicle may protect both arterial perforator branches and accompanying venous channels. Reported risk-factor analyses and venous-supercharging studies indicate that venous congestion and partial necrosis remain important issues in lower-extremity perforator or propeller flap reconstruction (23, 24). This may help explain why no venous congestion or marginal necrosis was recorded in this series, although the small sample size prevents firm conclusions about complication rates. Future studies using indocyanine green angiography, duplex ultrasound, or other perfusion-assessment methods would help determine how reliably this broad-pedicle design enhances arterial inflow and venous drainage.

In practical terms, the technique sits between a conventional local fasciocutaneous flap and a highly dissected single-perforator propeller flap. It is more anatomically directed than a random local flap because the axis and pedicle are planned along the posterior tibial artery perforator distribution. At the same time, it is less dependent on demanding perforator dissection than a single-perforator flap. This intermediate position may be especially relevant in hospitals where microsurgery is available only selectively or where complex infected lower-leg wounds require a relatively straightforward, vessel-preserving option. Nevertheless, the method still requires sound judgment regarding wound debridement, flap size, pedicle location, and the limits of local tissue transfer.

### Technical considerations

Several technical details are important for safe use of this flap. Preoperative handheld Doppler mapping is useful for identifying likely perforator locations and planning the flap axis. Although Doppler mapping cannot fully replace angiographic assessment, it provides a practical method for locating perforator signals along the medial tibial border. Safety data and technical reports on perforator propeller flaps emphasize the importance of source-vessel preservation, careful perforator selection, and avoidance of excessive torsion or tension (25–27). The pivot point should be chosen in healthy tissue adjacent to the wound whenever possible. In cases with extensive scar or infection, the surgeon should avoid designing the pedicle through the most compromised portion of the wound bed. The flap should be slightly larger than the defect to allow inset without tension, and the route of transfer should avoid compression or twisting of the fascial pedicle.

During elevation, preservation of a broad fascial pedicle is central to the technique. A 3–4 cm strip of deep fascia was retained in this study to carry serial perforators and small vascular branches. Published series of propeller or perforator-based flaps for middle and distal leg defects, including pediatric lower-extremity applications, further support the need for careful flap design and cautious patient selection (28–30). Excessive dissection around individual perforators may damage small vessels or provoke vasospasm and may undermine the rationale for the broad-pedicle design. For this reason, skeletonization was avoided. The flap was elevated in the subfascial plane, and the deep fascia was kept attached to the skin paddle. This approach is technically simpler than meticulous single-perforator dissection, but it still requires careful handling of the pedicle and attention to flap geometry.

Postoperative care is also important. Limb elevation reduces venous pressure and edema, while close monitoring during the first 48 h allows early detection of circulatory compromise. Although no vascular crisis occurred in this cohort, a tight tunnel or twisted pedicle could theoretically threaten flap perfusion. When the transfer route is narrow, opening the tunnel or extending the incision is preferable to forcing the flap through a constricted passage. In patients with infection history, adequate debridement and infection control before flap transfer are essential. Negative-pressure therapy and antibiotics were used when clinically indicated, but because detailed microbiological and treatment-duration data were not uniformly captured, these factors could not be analyzed quantitatively in the present study.

### Interpretation of clinical outcomes

Complete flap survival in all 15 patients is encouraging. The absence of partial necrosis suggests that the preserved serial perforator connections and broad fascial pedicle may provide adequate perfusion for the selected defect sizes treated in this cohort. However, this result should not be generalized to all lower-leg wounds. The study included selected patients whose wounds were appropriate for a local medial-leg flap, and the maximum wound size was approximately 5 × 8 cm. Larger circumferential defects, defects outside the reach of the medial posterior tibial artery perforator territory, or defects in limbs with major posterior tibial artery compromise may still require alternative reconstruction, including free tissue transfer.

Sensory recovery is another potential advantage of this flap, because the medial-leg vascular network lies near sensory nerve branches such as the saphenous nerve. In our cohort, 8 patients reached S3, 5 reached S2, and 2 reached S1. These results indicate that protective sensation may return in many patients, which is important for lower-leg and ankle coverage exposed to footwear, pressure, and repeated minor trauma. Nevertheless, the assessment was based on the MRC scale and retrospective chart review. Standardized quantitative sensory testing and patient-reported sensory outcomes were not available for all patients. Therefore, while sensory recovery was clinically meaningful, the present data cannot determine whether this flap provides superior sensory restoration compared with other flaps.

Ankle motion was preserved, with final dorsiflexion ranging from 15 degrees to 30 degrees and plantarflexion ranging from 30 degrees to 45 degrees. These ranges were clinically compatible with independent ambulation in all patients. The donor site did not cause recorded functional impairment, which is consistent with the fact that the technique harvests skin and fascia without sacrificing major muscle or the main posterior tibial artery. However, the lack of preoperative ankle measurements, contralateral comparison, gait analysis, and validated functional scores limits interpretation. Future studies should include standardized functional assessments such as ankle-hindfoot scoring, patient-reported outcome instruments, and objective gait or pressure analyses when feasible.

### Comparison with alternative reconstructive options

The posterior tibial artery perforator-based fasciocutaneous flap with a broad fascial pedicle should be viewed as one option within the reconstructive ladder rather than as a universal substitute for other flaps. Free flaps remain highly valuable for large, composite, infected, or poorly vascularized defects that exceed the reach or size of local flaps. Comparative studies have evaluated posterior tibial/peroneal perforator-plus flaps, propeller flaps, and free flaps for lower-extremity reconstruction, supporting individualized selection rather than a single preferred technique (31–33). Cross-leg and bridge-type reconstructive approaches may also remain useful in selected complex lower-extremity defects when local or ipsilateral options are unsuitable (34, 35).

For medial lower-leg and medial ankle wounds of moderate size, however, this posterior tibial artery perforator-based fasciocutaneous flap may have several potential advantages. It uses tissue from the same anatomical region, avoids microsurgical anastomosis, preserves the posterior tibial artery main trunk, and may include sensory nerve branches. In acute trauma and other selected lower-extremity defects, propeller and perforator-based local flaps have been reported as useful reconstructive options when carefully indicated (36). Other refined perforator-based strategies, including superthin flaps, adipofascial/ADM-assisted approaches, and posterior tibial artery perforator V-Y advancement flaps, have also been reported for selected extremity or lower-limb defects (37–39). These anatomical and practical considerations support the use of the technique in carefully selected cases, while not proving superiority over other approaches.

The current results are consistent with the concept that local perforator-based flaps can be effective for lower-leg reconstruction when the indications are appropriate. At the same time, the absence of a control group prevents direct comparison with sural flaps, peroneal perforator flaps, free flaps, or single posterior tibial artery perforator flaps. Statements implying that this technique is superior to other methods were therefore avoided in the revised manuscript. A more balanced interpretation is that a posterior tibial artery perforator-based fasciocutaneous flap with a broad fascial pedicle may provide reliable coverage for selected complex medial or distal lower-leg wounds, especially when the posterior tibial artery and adjacent perforators are clinically suitable and when a pedicled option is preferred.

### Clinical indications and boundary conditions

Based on the present experience, the most appropriate indication for a posterior tibial artery perforator-based fasciocutaneous flap with a broad fascial pedicle appears to be a moderate-sized medial or distal lower-leg defect with exposed deep structures, after adequate debridement and when adjacent medial-leg tissue remains available. The technique may be particularly suitable when the defect lies near the posterior tibial artery perforator axis, when a pedicled flap is preferred, and when preservation of major axial vessels is important. It may also be useful when previous infection or orthopedic surgery makes recipient-vessel preparation for free flap transfer less attractive.

The technique also has important boundaries. It should not be selected simply because it is technically simpler than free tissue transfer. If the wound is too large, if the defect is circumferential, if local tissue has been severely damaged, or if the posterior tibial artery territory is clinically unreliable, another reconstruction may be safer. Similarly, uncontrolled infection, inadequate debridement, or excessive pedicle tension may compromise results regardless of flap choice. These considerations were emphasized in the revised manuscript to guide appropriate use and to avoid implying that the flap is universally applicable.

### Study limitations

This study has several limitations. First, it was retrospective and conducted at a single center. Second, the sample size was small, with only 15 patients, which limits statistical power and external validity. Third, no control group was included; therefore, the study cannot determine whether this posterior tibial artery perforator-based fasciocutaneous flap with a broad fascial pedicle is superior to sural flaps, peroneal perforator flaps, free flaps, or single-perforator flaps. Fourth, patient selection bias is likely, because only patients considered suitable for this local flap were treated with the technique. The absence of venous congestion or partial necrosis should therefore be interpreted cautiously; it may reflect careful patient selection, moderate defect size, and avoidance of cases with unsuitable local tissue or unreliable perforator signals rather than a generalizable absence of risk. Fifth, wound etiology was heterogeneous, including post-traumatic defects and chronic post-surgical wounds after osteomyelitis debridement. Sixth, preoperative vascular assessment was based mainly on clinical evaluation and handheld Doppler mapping, and standardized angiographic or duplex data were not available for all patients. Seventh, functional and sensory outcomes were assessed descriptively, without validated patient-reported outcome measures or uniform objective testing. Although not all potentially relevant perioperative and follow-up variables were uniformly available in the original records, the core de-identified patient-level baseline, operative, and outcome data that could be verified from the source records are provided in Supplementary Table S1. Another limitation is that complete serial photographic documentation suitable for publication was available for only one representative case. Therefore, the photographic presentation may not fully capture the heterogeneity of the entire cohort. Detailed microbiological variables, standardized vascular imaging, validated functional scores, and patient-reported outcomes were still not uniformly available and therefore remain limitations of the study. Finally, follow-up ranged from 6 to 24 months, which may be insufficient to identify late breakdown, recurrent ulceration, scar contracture, or long-term sensory changes. These limitations mean that the findings should be considered preliminary and clinically hypothesis-generating.

## Conclusion

In this Original Research article based on a retrospective single-center clinical study, a posterior tibial artery perforator-based fasciocutaneous flap with a broad fascial pedicle achieved complete survival in 15 selected patients with complex medial or distal lower-leg wounds. The technique provided durable coverage, preserved independent ambulation, and was associated with minimal donor-site morbidity in this cohort. Its potential advantages include preservation of the posterior tibial artery main trunk, use of adjacent medial-leg tissue, avoidance of microsurgical anastomosis, and possible sensory recovery. However, because the study was retrospective, uncontrolled, small, and limited by photographic documentation from one representative case, the results should not be interpreted as evidence that the technique is superior to other reconstructive methods. Further prospective, multi-center, and comparative studies with standardized vascular, functional, and patient-reported outcome assessments are needed to clarify the indications and relative value of this flap.

## Data Availability

The de-identified data supporting the conclusions of this article will be made available by the corresponding author upon reasonable request, subject to institutional, ethical, and privacy restrictions.
