# Supplementary material for: Application of a posterior tibial artery perforator-based fasciocutaneous flap with a broad fascial pedicle in reconstruction of complex lower-leg wounds: a retrospective clinical study
Source: Front Surg. 2026 Jun 24;13:1849803. doi: 10.3389/fsurg.2026.1849803 (PMC13341658; doi:10.3389/fsurg.2026.1849803)
Supplement: Supplementary file 1 [file Table1.docx]

**Supplementary Table S1. De-identified patient-level clinical characteristics and outcomes**

**Supplementary Table S1A. Baseline and wound characteristics.**

| **Patient No.** | **Sex** | **Age** | **Etiology** | **Wound location** | **Wound size (cm)** | **Exposed structure** | **History of infection/prior surgery** | **Preoperative wound management** |
| --- | --- | --- | --- | --- | --- | --- | --- | --- |
| 1 | Male | 45 | Chronic post-surgical wound after osteomyelitis debridement | Medial ankle | 4.0 × 5.0 | Tibia + internal fixation material | Yes | Debridement + antibiotics and/or NPWT as indicated |
| 2 | Male | 32 | Post-traumatic soft-tissue loss | Middle-to-distal medial lower leg | 3.5 × 6.0 | Tibia + tendon | No | Routine optimization as indicated |
| 3 | Female | 58 | Chronic post-surgical wound after osteomyelitis debridement | Distal medial lower leg | 5.0 × 8.0 | Tibia | Yes | Debridement + antibiotics and/or NPWT as indicated |
| 4 | Male | 28 | Post-traumatic soft-tissue loss | Above medial malleolus | 3.0 × 4.5 | Tendon and/or bone | No | Routine optimization as indicated |
| 5 | Male | 59 | Chronic post-surgical wound after osteomyelitis debridement | Medial ankle | 4.5 × 5.5 | Bone + internal fixation material | Yes | Debridement + antibiotics and/or NPWT as indicated |
| 6 | Female | 36 | Post-traumatic soft-tissue loss | Middle medial lower leg | 4.0 × 7.0 | Tibia | No | Routine optimization as indicated |
| 7 | Male | 51 | Chronic post-surgical wound after osteomyelitis debridement | Distal medial lower leg | 3.0 × 4.0 | Tibia | Yes | Debridement + antibiotics and/or NPWT as indicated |
| 8 | Male | 22 | Post-traumatic soft-tissue loss | Medial ankle | 2.5 × 3.5 | Medial malleolus/bone | No | Routine optimization as indicated |
| 9 | Female | 49 | Chronic post-surgical wound after osteomyelitis debridement | Middle-to-distal medial lower leg | 5.0 × 6.5 | Tibia + internal fixation material | Yes | Debridement + antibiotics and/or NPWT as indicated |
| 10 | Male | 39 | Post-traumatic soft-tissue loss | Upper-to-middle medial lower leg | 3.5 × 5.0 | Tibia | No | Routine optimization as indicated |
| 11 | Male | 65 | Chronic post-surgical wound after osteomyelitis debridement | Medial ankle | 4.0 × 6.0 | Bone | Yes | Debridement + antibiotics and/or NPWT as indicated |
| 12 | Male | 42 | Post-traumatic soft-tissue loss | Distal medial lower leg | 4.5 × 7.0 | Tibia + tendon | No | Routine optimization as indicated |
| 13 | Female | 33 | Post-traumatic soft-tissue loss | Above medial malleolus | 3.0 × 4.0 | Tendon | No | Routine optimization as indicated |
| 14 | Male | 53 | Chronic post-surgical wound after osteomyelitis debridement | Middle-to-distal medial lower leg | 3.5 × 5.5 | Tibia + internal fixation material | Yes | Debridement + antibiotics and/or NPWT as indicated |
| 15 | Male | 41 | Post-traumatic soft-tissue loss | Medial ankle | 3.0 × 4.5 | Bone + internal fixation material | Yes (prior surgery) | Routine optimization as indicated |

*Abbreviation: NPWT, negative-pressure wound therapy.*

**Supplementary Table S1B. Operative variables and postoperative outcomes.**

| **Patient No.** | **Flap size (cm)** | **Donor-site closure** | **Follow-up (months)** | **Flap survival** | **Partial/total necrosis** | **MRC sensory grade** | **Ankle dorsiflexion (degrees)** | **Ankle plantarflexion (degrees)** | **Postoperative complications** | **Revision surgery** |
| --- | --- | --- | --- | --- | --- | --- | --- | --- | --- | --- |
| 1 | 5.0 × 6.0 | Primary closure | 24 | Complete | None | S3 | 25 | 40 | None recorded | No |
| 2 | 4.0 × 7.0 | Primary closure | 18 | Complete | None | S3 | 28 | 42 | None recorded | No |
| 3 | 6.0 × 9.0 | STSG | 12 | Complete | None | S2 | 20 | 35 | None recorded | No |
| 4 | 3.5 × 5.5 | Primary closure | 15 | Complete | None | S3 | 30 | 45 | None recorded | No |
| 5 | 5.5 × 6.5 | Primary closure | 9 | Complete | None | S2 | 18 | 32 | None recorded | No |
| 6 | 5.0 × 8.0 | STSG | 21 | Complete | None | S3 | 22 | 38 | None recorded | No |
| 7 | 4.0 × 5.0 | Primary closure | 14 | Complete | None | S2 | 24 | 40 | None recorded | No |
| 8 | 3.0 × 4.5 | Primary closure | 6 | Complete | None | S1 | 26 | 42 | None recorded | No |
| 9 | 6.0 × 7.5 | STSG | 16 | Complete | None | S3 | 15 | 30 | None recorded | No |
| 10 | 4.5 × 6.0 | Primary closure | 20 | Complete | None | S3 | 23 | 36 | None recorded | No |
| 11 | 5.0 × 7.0 | STSG | 8 | Complete | None | S1 | 17 | 33 | None recorded | No |
| 12 | 5.5 × 8.0 | STSG | 13 | Complete | None | S2 | 21 | 37 | None recorded | No |
| 13 | 3.5 × 5.0 | Primary closure | 11 | Complete | None | S2 | 27 | 43 | None recorded | No |
| 14 | 4.5 × 6.5 | Primary closure | 17 | Complete | None | S3 | 19 | 34 | None recorded | No |
| 15 | 3.5 × 5.5 | Primary closure | 10 | Complete | None | S3 | 25 | 41 | None recorded | No |

*Abbreviations: MRC, Medical Research Council; STSG, split-thickness skin graft.*
